# The Proteasome System in Infection: Impact of β5 and LMP7 on Composition, Maturation and Quantity of Active Proteasome Complexes

**DOI:** 10.1371/journal.pone.0039827

**Published:** 2012-06-29

**Authors:** Thorsten Joeris, Nicole Schmidt, David Ermert, Petra Krienke, Alexander Visekruna, Ulrike Kuckelkorn, Stefan H. E. Kaufmann, Ulrich Steinhoff

**Affiliations:** 1 Department of Immunology, Max Planck Institute for Infection Biology, Berlin, Germany; 2 Department of Cellular Microbiology, Max Planck Institute for Infection Biology, Berlin, Germany; 3 Institute for Microbiology and Hygiene, Philipps University Marburg, Hessen, Marburg, Germany; 4 Institute for Biochemistry, Charité Berlin, Berlin, Germany; University of London, St George's, United Kingdom

## Abstract

Proteasomes are the major enzyme complexes for non-lysosomal protein degradation in eukaryotic cells. Mammals express two sets of catalytic subunits: the constitutive subunits β1, β2 and β5 and the immunosubunits LMP2 (β1i), MECL-1 (β2i) and LMP7 (β5i). The LMP7-propeptide (proLMP7) is required for optimal maturation of LMP2/MECL-1-containing precursors to mature immunoproteasomes, but can also mediate efficient integration into mixed proteasomes containing β1 and β2. In contrast, the β5-propeptide (proβ5) has been suggested to promote preferential integration into β1/β2-containing precursors, consequently favouring the formation of constitutive proteasomes. Here, we show that proβ5 predominantly promotes integration into LMP2/MECL-1-containing precursors in IFNγ-stimulated, LMP7-deficient cells and infected LMP7-deficient mice. This demonstrates that proβ5 does not direct preferential integration into β1/β2-containing precursors, but instead promotes the formation of mixed LMP2/MECL-1/β5 proteasomes under inflammatory conditions. Moreover, the propeptides substantially differ in their capacity to promote proteasome maturation, with proLMP7 showing a significantly higher chaperone activity as compared to proβ5. Increased efficiency of proteasome maturation mediated by proLMP7 is required for optimal MHC class I cell surface expression and is equally important as the catalytic activity of immunoproteasomes. Intriguingly, induction of LMP7 by infection not only results in rapid exchange of constitutive by immunosubunits, as previously suggested, but also increases the total proteasome abundance within the infected tissue. Hence our data identify a novel LMP7-dependend mechanism to enhance the activity of the proteasome system in infection, which is based on the high chaperone activity of proLMP7 and relies on accelerated maturation of active proteasome complexes.

## Introduction

Proteasomes execute the majority of non-lysosomal protein degradation in eukaryotic cells [1]. The central 20S complex of the proteasome is a barrel-like structure composed of α- and β-subunits, which form heptameric rings arranged in an α_1–7_β_1–7_β_1–7_α_1–7_ stoichiometry [2]. The assembly of 20S proteasomes is a well-ordered process. At first the proteasome assembling chaperones (PAC1-4) form a scaffold for the organisation of an α-ring, which subsequently serves as a matrix for binding of the β-subunits, resulting in the formation of 13-15S precursor proteasomes [3], [4], [5]. Finally, the proteasome maturation protein (POMP) assists the assembly of two 15S half proteasomes to mature 20S complexes [6], [7]. During the assembly process the propeptides of β-subunits exert a chaperone-like function, which is required for the effective maturation of precursor proteasomes [6], [8]. Completion of 20S proteasome assembly is accompanied by autocatalytic removal of the propeptides, which activates the catalytic sites of the mature β-subunits [9].

The 20S proteasome has three major proteolytic activities defined as caspase-, trypsin- and chymotrypsin-like [2], with the corresponding catalytic sites being assigned to the constitutive subunits β1 (Y), β2 (Z) and β5 (X), respectively [10]. In mammals, the proteasome system displays further plasticity as three interferon-gamma (IFNγ-inducible β-subunits, the immunosubunits LMP2 (β1i), MECL-1 (β2i) and LMP7 (β5i), can replace their constitutive counterparts. Integration of either constitutive or immunosubunits gives rise to the major 20S proteasome subsets, named constitutive and immunoproteasomes respectively [11].

In the immune system proteasomes are the major source of antigenic peptides presented on major histocompatibility complex (MHC) class I molecules [1] and induction of immunoproteasomes is known to optimize this process [11]. Deletion of LMP7, but not LMP2 or MECL-1, reduces MHC class I cell surface expression by about 25–50% [11], [12], demonstrating the unique importance of this subunit for optimal antigen presentation.

In addition, LMP7 containing proteasomes have been described to drive inflammatory responses, which can in part be attributed to enhanced activation of the transcription factor NF-κB [13], [14], [15] and expression of LMP7 was shown to be crucial for resistance against oxidative stress [16]. At present, these diverse functions of LMP7 are solely attributed to its specific proteolytic activity [11], [15], [16]. However, besides its catalytic activity, expression of LMP7 has a substantial impact on the assembly and composition of 20S proteasomes. In this context, it has been shown that expression of LMP7 accelerates the rate of proteasome assembly, supporting the rapid formation of immunoproteasomes in infection and inflammation [17]. Further, it has been described that efficient maturation of LMP2/MECL-1-containing precursor proteasomes requires the propeptide of LMP7 (proLMP7), a concept known as cooperative assembly of immunoproteasomes [18], [19]. However, integration of LMP7 is not restricted to immunoproteasomes, since proLMP7 also mediates integration into proteasomes containing β1 and β2 [19]. In line with this, various combinations of mixed proteasomes were recently identified in human tissues and cell lines [20], [21]. In contrast to proLMP7, the propeptide of β5 (proβ5) has been suggested to favour integration into β1/β2-containing precursors and thus formation of mixed proteasomes with LMP2/MECL-1/β5 stoichiometry is supposed to be a rare event [18], [19]. Despite this extensive knowledge on the impact of LMP7 and β5 on proteasome assembly and composition, it is still not clear how the structural functions of these two subunits influence the proteasome system in infection.

Here, we analyzed the function of proLMP7 and proβ5 in reconstituted LMP7-deficient murine embryonic fibroblasts (*lmp7^−/−^* Mefs) and mimicked inflammatory conditions by IFNγ stimulation. We found that not only proLMP7, but also proβ5 mediates predominant integration into LMP2/MECL-1-containing precursor proteasomes following IFNγstimulation, leading to considerable formation of mixed proteasomes with LMP2/MECL-1/β5 stoichiometry. High abundance of such mixed proteasomes was also detected following infection of *lmp7^−/−^* mice, confirming that proβ5 does not have a preference for β1/β2-containing precursors, but can also generate mixed proteasomes. The propeptides however differed significantly in their capacity to promote proteasome maturation under inflammatory conditions, with proLMP7 showing a significantly higher efficiency to promote this process as compared to proβ5. In infection, accelerated proteasome maturation driven by LMP7 did not only result in a rapid exchange of constitutive by immunoproteasomes as previously suggested [17], [22], but also in increased total proteasome quantity within the infected tissue. The specific proteolytic activity of LMP7 was however not crucial for enhanced proteasome maturation, since the chimeric proLMP7mβ5 protein had a similar capacity to promote this process as compared to full-length LMP7. This identifies proLMP7 as the critical pacemaker, which accelerates the maturation of proteasomes under inflammatory conditions. Thus, we delineate a novel mechanism of LMP7-dependent regulation of the proteasome system in infection, which increases the proteasomal activity by enhanced generation of mature proteasome complexes.

## Results

### proLMP7 and proβ5 mediate incorporation into LMP2/MECL-1-containing precursor proteasomes upon IFNγstimulation

**Figure 1:**
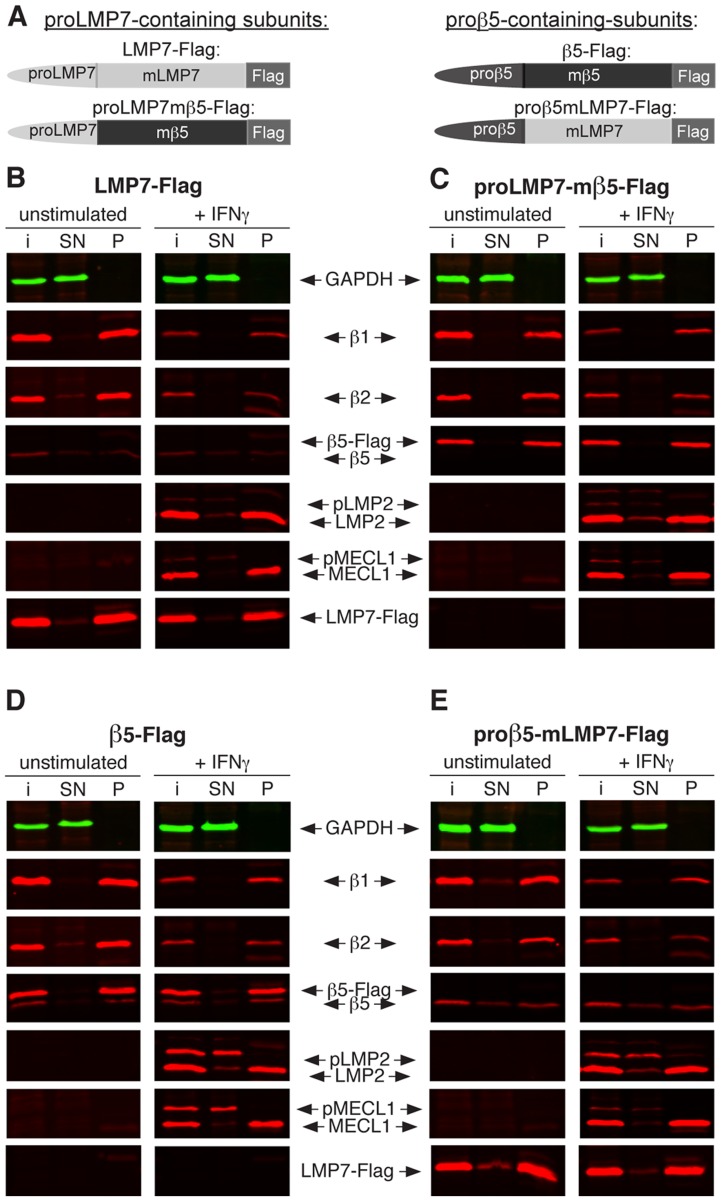
Co-immunoprecipitation analysis using proβ5- and LMP7-containing proteasome subunits over-expressed in *lmp7* *^−^* ^*/−*^Mefs. Flag-tagged full-length LMP7 (LMP7-Flag), proLMP7 fused to mature β5 (proLMP7mβ5-Flag), full-length β5 (β5-Flag) and proβ5 fused to mature LMP7 (proβ5mLMP7-Flag) were over-expressed in *lmp7^−/−^*Mefs by retroviral transduction (A). The *lmp7^−/−^* Mef lines expressing the four different constructs were either left unstimulated or cultured in the presence of 50 U/ml IFNγ for 4 days. Following cell lysis, the Flag-tagged subunits were precipitated with anti-Flag-M2® agarose. Co-precipitation of the catalytic proteasome subunits β1, β2, LMP2 and MECL-1 with the proLMP7-containing subunits LMP7-Flag (B) and proLMP7mβ5-Flag (C) or the proβ5-containing subunits β5-Flag (D) and proβ5mLMP7-Flag (E), was analysed by Two-colour fluorescent immunoblot analysis. The abundance of each subunit was determined in the input material (i), the supernatant of the immunoprecipitation (SN) and the precipitate (P) for both conditions tested.

To study the function of proβ5 and proLMP7 under homeostatic and inflammatory conditions, we generated LMP7-deficient murine embryonic fibroblasts (*lmp7* ^−/−^ Mefs), which were reconstituted with Flag-tagged constructs of either full-length LMP7 (LMP7-Flag), proLMP7 fused to mature β5 (proLMP7mβ5-Flag), full-length β5 (β5-Flag) or proβ5 fused to mature LMP7 (proβ5mLMP7-Flag) (Fig. 1A). The reconstituted *lmp7^−/−^* Mefs displayed high expression of β1 and β2 during homeostasis and high amounts of LMP2 and MECL-1 after IFNγstimulation, which mimicked inflammatory conditions (Fig. 1B–E). The anti-Flag antibody did not precipitate proteasomes in *lmp7^−/−^* Mefs transduced with the empty vector construct (Fig. S1A), verifying that the precipitation was specific for flag-tagged complexes. Immunoprecipitation of the proLMP7-containing subunits, LMP7-Flag and proLMP7mβ5-Flag, cleared the supernatants of all catalytic proteasome subunits in unstimulated and IFNγ treated cells, demonstrating that proLMP7 mediates effective integration into all types of proteasomes, present under both conditions (Fig. 1B–C). Complete co-precipitation of β1 and β2 was also observed in unstimulated *lmp7^−/−^* Mefs expressing the proβ5-containing subunits, β5-Flag or proβ5mLMP7-Flag (Fig. 1D–E), demonstrating effective maturation of β1/β2-containing precursors as expected. However, following IFNγ stimulation, LMP2 and MECL-1 were efficiently co-precipitated with proβ5-containing subunits (Fig. 1D–E), revealing that proβ5 can also mediate substantial maturation of LMP2/MECL-1-containing precursors. Simultaneously, the abundance of β1 and β2 was reduced upon IFNγ stimulation (Fig. 1B–E, Fig. S1A), suggesting that the proβ5-containing subunits did not favour integration into proteasomes containing β1 and β2, but integrated into LMP2/MECL-1-containing precursors instead.

To assess, whether β5 is capable to integrate into LMP2/MECL-1-containing precursors in the presence of LMP7, β5-Flag was over-expressed in wild type Mefs (WT-Mefs). In this setting, β5-Flag also co-precipitated with LMP2 and MECL-1 following IFNγ stimulation, while the amount of co-precipitated β1 and β2 was reduced (Fig. S1B). This confirms that β5 can integrate into LMP2/MECL-1-containing precursors, even in competition with LMP7.

Still, low levels of unprocessed pLMP2 and pMECL-1 were only detected in supernatants of *lmp7^−/−^* Mefs reconstituted with the proβ5-containing subunits (Fig. 1D–E), revealing that proβ5 is a limiting factor for maturation of proteasomes under inflammatory conditions. However, our data indicate that this is not due to a preference of proβ5 for β1/β2-containing precursors as suggested previously [19]. Instead, it appears that proβ5 displays a generally lower capacity to promote proteasome maturation, which subsequently becomes a limiting factor for the maturation of proteasomes under inflammatory conditions.

### proLMP7 mediates higher efficiency of proteasome maturation compared to proβ5

It has been suggested that accelerated proteasome maturation by LMP7 is a function of its propeptide, since proLMP7 shows high affinity to the maturation factor POMP [17]. However, direct experimental evidence that proLMP7 mediates accelerated proteasome maturation is missing. Thus it remains unclear, whether only the propeptide or also the specific proteolytic activity and/or the carboxy-terminus of LMP7 are involved in this process. To address this issue, we analysed proteasome maturation in the reconstituted *lmp7^−/−^* Mefs.

**Figure 2:**
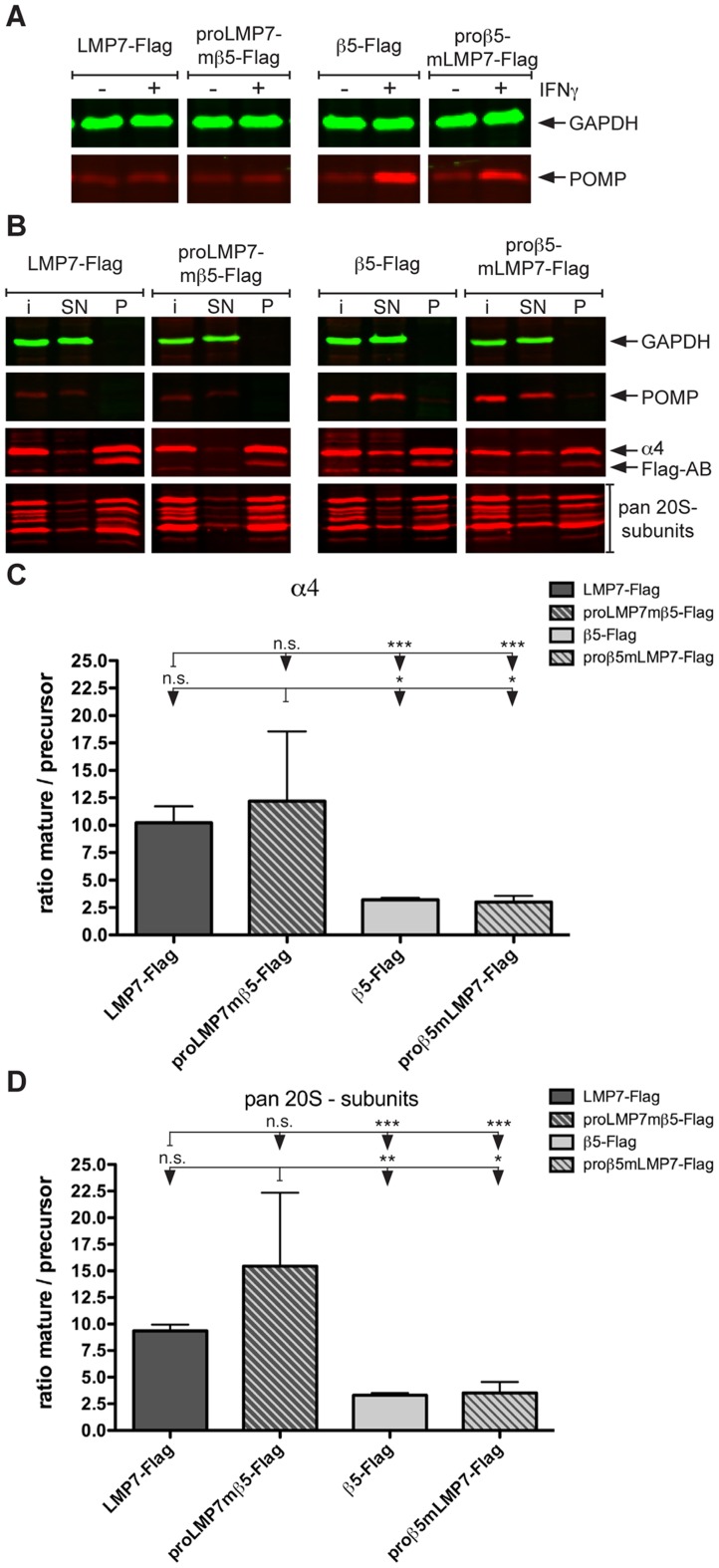
Relative quantification of proteasome maturation in *lmp7* *^−^* ^***/****−*^ **Mefs expressing proLMP7- or proβ5-containing subunits.** (A) *lmp7^−/−^* Mefs expressing the proLMP7-containing subunits LMP7-Flag and proLMP7mβ5-Flag or the proβ5-containing subunits β5-Flag and proβ5mLMP7-Flag were either left unstimulated or cultured in the presence of 50 U/ml IFNγ for 4 days. The abundance of POMP in the cell lysates of the four cell lines was determined by immunoblot analysis. (B) Immunoprecipitation was performed with anti-Flag M2® agarose using cell lysates of the four different *lmp7^−/−^* Mef lines, which were grown in the presence of 50 U/ml IFNγ for 4 days. The abundance of GAPDH, POMP, α4 and pan-20S proteasome subunits (pan 20S subunits) was determined by immunoblot analysis of the input material (i), the supernatants (SN) and precipitates (P) following immunoprecipitation with anti-Flag M2® agarose. (C, D) The ratio mature/precursor resembles the abundance of α4 subunits (C) or pan 20S subunits (D) integrated into mature proteasomes divided by their abundance in precursor proteasomes and was calculated as follows: band intensity of subunit X in the precipitate (P) divided by the band intensity of subunit X in the supernatant (SN). Given values for each cell line are mean values ± standard deviations of at least three independent experiments.

The maturation factor POMP was used as an indicator for the presence of precursor proteasomes, since it is found in 13-15S precursors, but not in mature complexes [6], [7]. POMP was exclusively detected in IFNγ-treated *lmp7^−/−^* Mefs reconstituted with the proβ5-containing subunits, β5-Flag or proβ5mLMP7-Flag (Fig. 2A), confirming that proβ5 limits proteasome-maturation specifically under inflammatory conditions. When IFNγ-stimulated, reconstituted *lmp7^−/−^* Mefs were analysed by immunoprecipitation, POMP was exclusively detected in the supernatants, but not the precipitates (Fig. 2B), demonstrating separation of mature proteasomes and the precursor fraction (Fig. 2B). This enabled us to determine the relative abundance of mature vs. precursor proteasomes in the reconstituted *lmp7^−/−^* Mefs. The abundance of α4 was used as correlate for the quantity of mature and precursor proteasomes, since this structural subunit is integrated in both fractions. The efficiency of proteasome maturation was expressed as ratio of α4-abundance in mature vs. precursor proteasomes. Accordingly, high ratios indicate effective maturation, while low ratios reflect accumulation of precursors and thus low maturation efficiency (Fig. 2C). LMP7-Flag and proLMP7mβ5-Flag showed no significant difference in the ratios of mature vs. precursor proteasomes, revealing that the proLMP7-containing subunits are equally efficient in driving proteasome maturation (Fig. 2C). Similarly, β5-Flag or proβ5mLMP7-Flag did not show a significant difference in their ratios; demonstrating that the proβ5-containing subunits also have a similar capacity to mediate proteasome maturation (Fig. 2C). These results were confirmed by staining with a polyclonal antiserum recognizing various α- and βsubunits (pan-20S-subunits, Fig. 2B, D).

It is well known that proteasome maturation requires the catalytic activity of the β-subunits for autocatalytic removal of the propeptides and for degradation of POMP [6], [9]. However, the efficiency of proteasome maturation observed for full-length LMP7 and the chimeric proLMP7mβ5 protein did not differ and the same holds true for full-length β5 and the chimeric proβ5mLMP7 protein (Fig. 2C, D). Hence, the specific proteolytic activity of the fused catalytic subunit is of minor importance for the efficiency of proteasome maturation, since it is not impaired as long as the proteolytic activity of either LMP7 or β5 is present. Accordingly, the propeptides are the crucial protein domains, which regulate the efficiency of proteasome maturation, whereas the proteolytic activities of the respective subunits or their carboxy-termini have no significant impact on this process.

Intriguingly, the ratios of mature vs. precursor proteasomes for the proLMP7-containing subunits were 3 to 4-fold higher as compared to the proβ5-containing subunits (Fig. 2C–D), demonstrating that proLMP7 has a substantially higher capacity to promote proteasome maturation as compared to proβ5. This confirms that accelerated proteasome maturation mediated by LMP7 is an exclusive function of its propeptide, as previously proposed [17]. Moreover, this finding underlines that the chaperone-function of proβ5 might become a limiting factor for proteasome maturation under inflammatory conditions.

### Optimal MHC class I expression requires both, catalytic activity of LMP7 and efficient proteasome maturation by proLMP7

**Figure 3:**
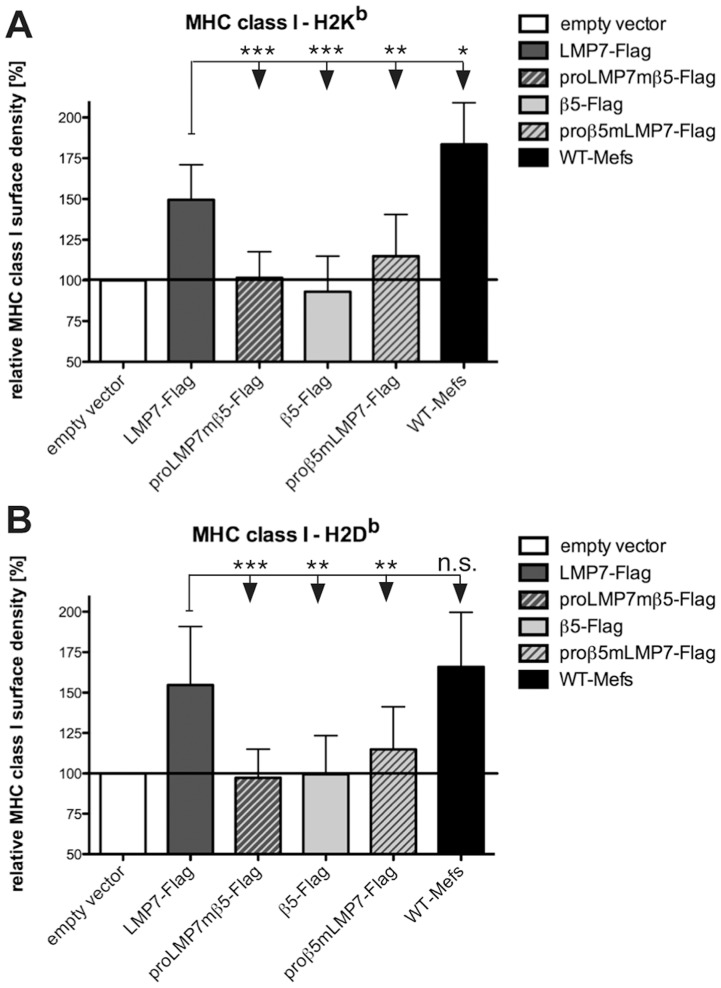
Analysis of MHC class I cell surface expression in *lmp7* *^−^* ^***/****−*^ **Mefs expressing proLMP7- or proβ5-containing subunits.** *lmp7^−/−^* Mefs expressing LMP7-Flag, proLMP7mβ5-Flag, β5-Flag, proβ5mLMP7-Flag or cells transduced with the empty vector construct were cultured in the presence of 50 U/ml IFNγ for 4 days. WT-Mefs stimulated with IFNγ for 4 days were used as a positive control. The MHC class I cell surface expression of the haplotypes H-2K^b^ (E) and H-2D^b^ (F) on the various cell lines was determined by flow cytometry. The relative MHC class I density of the different cell lines was calculated by normalization of the median fluorescence intensity (MFI) of the indicated cell line to the MFI of *lmp7^−/−^* Mefs transduced with the empty vector construct. Given values are means ± standard deviations of five independent experiments.

Deletion of LMP7 results in a significant decrease in MHC class I cell surface expression [12], but it is not known whether this phenotype is entirely caused by a lack of proteolytic activity of LMP7 or also affected by impaired proteasome maturation. Since proteasome maturation was restored in *lmp7^−/−^* Mefs expressing proLMP7mβ5-Flag (Fig. 2A–D), we wondered whether the formation of mixed LMP2/MECL-1/β5 proteasomes, was also capable of rescuing MHC class I cell surface expression. However, in contrast to reconstitution with LMP7-Flag, expression of proLMP7mβ5-Flag did not rescue surface expression of the MHC I molecules H2K^b^ and H2D^b^ (Fig. 3A–B). Accordingly, even strong formation of mixed LMP2/MECL-1/β5 proteasomes in *lmp7^−/−^* Mefs expressing proLMP7mβ5-Flag (Fig. 1C) could not substitute for the formation of immunoproteasomes, demonstrating that the proteolytic activity of LMP7 is indispensable for optimal MHC class I cell surface expression. Nevertheless, surface expression of H2K^b^ and H2D^b^ was also impaired in *lmp7^−/−^* Mefs reconstituted with proβ5mLMP7-Flag (Fig. 3A–B), but since considerable amounts of immunoproteasomes were detected in these cells after IFNγ stimulation (Fig. 1E), an increase in MHC class I surface expression would have been expected. Accordingly, the fusion of proβ5 to LMP7 also limits the MHC class I cell surface expression. This reveals that optimal MHC class I cell surface expression does not only require the proteolytic activity of LMP7, but furthermore requires the chaperone activity of proLMP7 to drive optimal proteasome maturation.

### Infection of lmp7*^−/−^* mice with listeriae induces formation of mixed proteasomes

The formation of mixed proteasomes containing LMP2, MECL-1 and β5 has been suggested to be inefficient due to preferential integration of β5 into constitutive proteasomes [18], [19]. Since we predominantly found LMP2/MECL-1/β5 proteasomes in IFNγ-stimulated *lmp7* ^−/−^Mefs *in vitro*, we wondered to which extent these mixed proteasomes were formed following infection of *lmp7^−/−^* mice *in vivo*. To this end, wild type control mice (WT mice) and *lmp7^−/−^* mice were infected with *Listeria monocytogenes*, which is known to trigger IFNγ-dependent expression of immunosubunits [22], [23]. 2D Blue Native/SDS-PAGE was used to identify various mature and precursor proteasome complexes (Fig. S2), which allowed to track the integration of catalytic proteasome subunits into diverse proteasome complexes.

As expected listeria-infection of WT mice induced increased formation of immunoproteasomes in the liver (Fig. S3A), while their abundance was constitutively high in spleen (Fig. S3B). Noteworthy, high abundance of LMP2 and LMP7 but not MECL-1 was detected in mature proteasomes of naïve WT liver (Fig. S3A), indicating the formation of LMP2/β2/LMP7 or β1/β2/LMP7 proteasomes, which were recently also identified in various human tissues [20].

**Figure 4:**
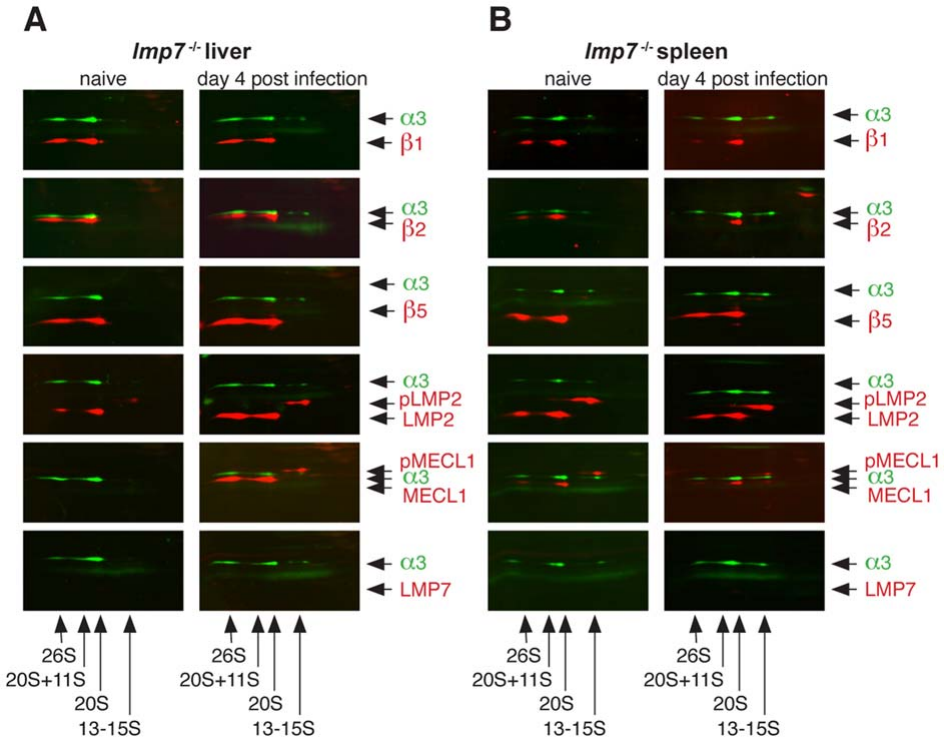
Analysis of proteasome composition in naive and listeria-infected *lmp7* *^−^* ^***/****−*^ **mice.** Organ lysates of liver (A) and spleen (B) of naïve and infected *lmp7^−/−^* mice were analysed by 2D two-colour fluorescent immunoblot analysis. The mice were infected i.v. with 5×10^3^ cfu *L. monocytogenes* and sacrificed 4 days post infection. Organs of three to four mice per group were pooled for the analysis. The different proteasome complexes present in the tissues were first separated by Blue Native-PAGE and subsequently by SDS-PAGE followed by two-colour fluorescent immunoblot analysis. Each membrane was stained for proteasome subunit α3 (green signal) and as it is present in all early to mature complexes in proteasome assembly, α3 serves as a marker for the presence and positions of 13-15S precursor proteasomes, 20S proteasomes and 20S proteasomes + 11S and 19S regulators. Further, membranes were stained for the indicated catalytic β-subunits (red signals) to identify, in which of the indicated proteasome complexes they were integrated.

Interestingly, LMP2 but not MECL-1 was detected in mature proteasomes of naïve *lmp7^−/−^* liver, revealing the formation of LMP2/β2/β5 proteasomes, which have not been described yet (Fig. 4A). Infection induced the integration of both, LMP2 and MECL-1, into mature proteasomes in *lmp7^−/−^* liver, confirming the formation of LMP2/MECL-1/β5 proteasomes *in vivo* (Fig. 4A). LMP2/MECL-1/β5 proteasomes were also detected in spleens of naïve and infected *lmp7^−/−^* mice, demonstrating constitutive formation of mixed proteasomes in the spleen. This is in line with the constitutively high expression of LMP2 and MECL-1 commonly found in lymphoid tissues [23]. Taken together this underlines that proβ5 does not only promote the formation of mixed LMP2/MECL-1/β5 *in vitro*, but also in infected or lymphoid tissue of *lmp7^−/−^* mice *in vivo*.

### Mixed LMP2/MECL-1/β5 proteasomes are the predominant proteasome type assembled following listeria-infection of lmp7*^−/−^* mice

**Figure 5:**
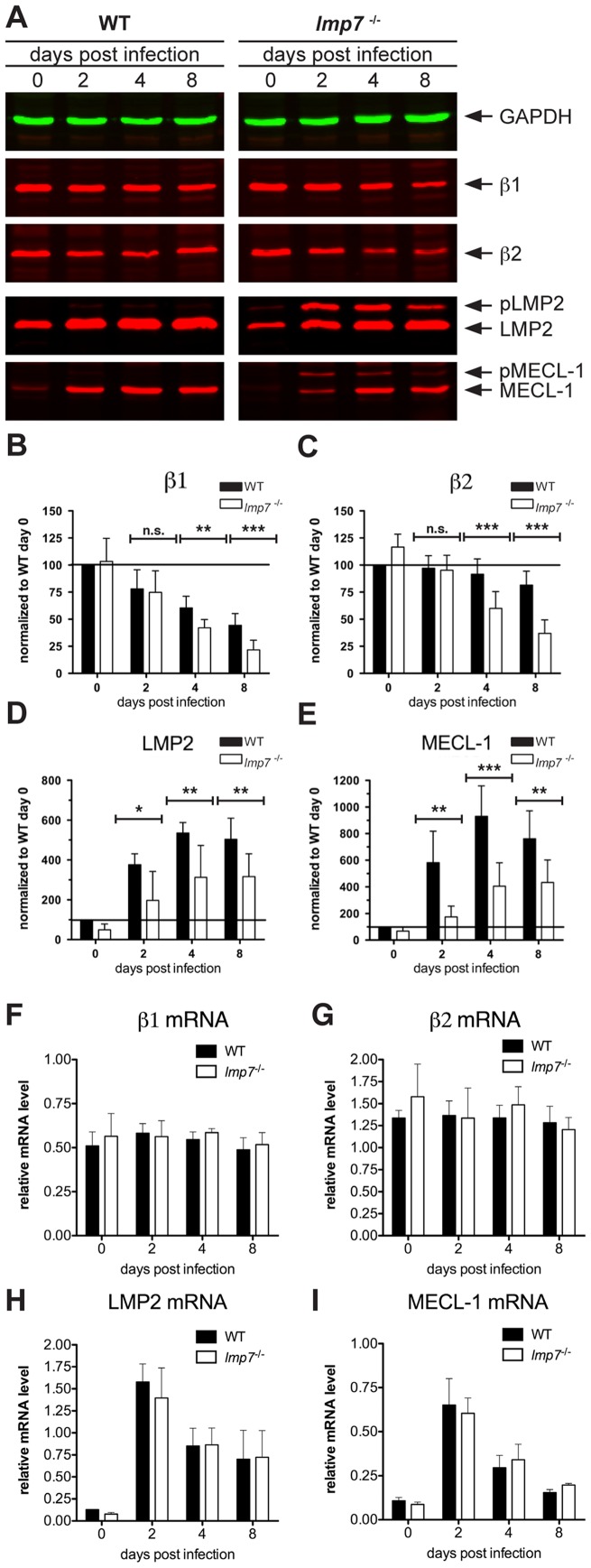
Quantification of β1, β2, LMP2 and MECL-1 expression at protein and mRNA level in WT and lmp7^−/−^ mice during the course of listeria infection. Both groups of mice were infected i.v. with 5×10^3^ cfu of *L. monocytogenes* and livers of three to four mice per group were pooled for two-colour fluorescent immunoblot analysis. Naïve mice (day 0) were used as controls in both groups of mice. Each membrane was stained against GAPDH as loading control. Further, membranes were stained with antibodies specific for β1, β2, LMP2 and MECL-1 as indicated. Following densitometric analysis the relative protein abundance of β1 (B), β2 (C), LMP2 (D) and MECL-1 (E) was expressed as band intensity normalized to WT day 0, which was calculated as follows: (band intensity of proteasome subunit X at day Y/band intensity of GAPDH at day Y)/(band intensity of proteasome subunit X in WT day 0/band intensity of GAPDH in WT day 0) ×100). The given results are means ± standard deviation of three independent infections and each sample was analysed in duplicates during immunoblot analysis (B–E). Total liver RNA of naïve and listeria-infected WT and *lmp7^−/−^* mice was isolated at the indicated time points and semi-quantitative qPCR analysis was performed. The relative mRNA expression of the proteasome subunits β1 (F), β2 (G), LMP2 (H) and MECL-1 (I) was calculated by normalization to the house keeping gene ribosomal protein subunit 9 (RPS9) using the ΔΔCT method. Each value represents mean ± standard deviation of three individual mice. Representative results of one of two independent infection experiments are shown.

Next, we aimed to quantify the abundance of constitutive and mixed LMP2/MECL-1/β5 proteasomes during the course of infection in *lmp7^−/−^* mice. In addition, we wanted to assess the maturation efficiency of LMP2/MECL-1-containing precursors in infected *lmp7^−/−^* compared to WT mice. For this purpose, the abundance of mature β1-β2-, LMP2- and MECL-1- subunits was determined by immunoblot analysis in mutant- and WT-mice (Fig. 5A). Following normalization we found that the abundance of β1andβ2 was substantially decreased at days 4 and 8 after infection in both groups of mice (Fig. 5 B–C). However, this drop in constitutive subunits was more pronounced in *lmp7^−/−^* as compared to WT mice (Fig. 5 B–C), indicating stronger replacement of constitutive proteasomes in infected *lmp7^−/−^* mice. Simultaneously, the abundance of mature LMP2 and MECL-1 was increased following listeria infection of *lmp7^−/−^* mice (Fig. 5 D–E), which is in line with the formation of LMP2/MECL-1/β5 proteasomes in infected *lmp7^−/−^* liver as shown in Fig. 4A. Similar results were obtained for the spleen of infected *lmp7^−/−^* mice, in which the abundance of β1andβ2 was also lower compared to infected WT mice, whereas the abundance of LMP2 and MECL-1 was increased by infection (Fig. S4A–E.) This strongly suggests that LMP2/MECL-1/β5 proteasomes were the predominant proteasome subtype assembled during listeria-infection of *lmp7^−/−^* mice and underlines that β5 does not have a preference for integration into constitutive proteasomes in infection.

Still, maturation of LMP2 and MECL-1 was less efficient in *lmp7^−/−^* as compared to WT mice, as seen by accumulation of unprocessed LMP2 (pLMP2) and MECL-1 (pMECL-1) (Fig. 5A) and significantly lower levels of mature LMP2 and MECL-1 (Fig. 5D–E). Taken together, this suggests that although β5 does not seem to have a preference for integration into constitutive proteasomes, it is still less efficient in mediating proteasome maturation in infection. This is most likely due to the low chaperone activity of proβ5, which we identified as a limiting factor for proteasome maturation in IFNγ stimulated *lmp7^−/−^* Mefs (Fig. 2).

Noteworthy, the mRNA expression levels of all analysed subunits did not differ between both groups of mice throughout the course of infection (Fig. 5F–I; Fig. S4F–I), ruling out that the differences in protein abundance, which were observed between both groups of mice (Fig. 5A–E; Fig. S4A–E), were due to differential mRNA expression.

### Infection induces an LMP7-dependent increase in proteasome quantity

**Figure 6:**
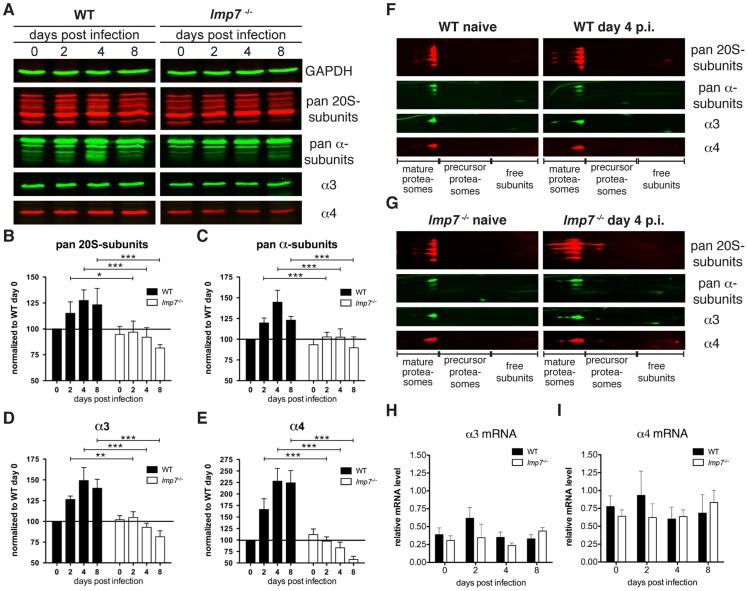
Relative quantification of mature proteasomes in WT and *lmp7* *^−^* ^***/****−*^ **mice during the course of listeria infection.** WT and *lmp7^−/−^* mice were infected i.v. with 5×10^3^ cfu of *L. monocytogenes* and livers of three to four mice per group were pooled for two-colour fluorescent immunoblot analysis. Naïve mice (day 0) were used as controls in both groups of mice. Each membrane was stained against GAPDH as loading control. Further, membranes were stained with antibodies recognizing pan-20S subunits, pan-α-subunits, α3 and α4as indicated. Representative blots of three independent experiments are shown (A). Following densitometric analysis the relative protein abundance of pan-20S subunits (B), pan-α-subunits (C), α3 (D) and α4E was expressed as band intensities normalized to WT day 0, which was calculated as follows: (band intensity of proteasome subunit X at day Y/band intensity of GAPDH at day Y)/(band intensity of proteasome subunit X in WT day 0/band intensity of GAPDH in WT day 0) × 100). The given results are means ± standard deviation of three independent infection experiments and each sample was analysed in duplicates during immunoblot analysis (B–E). To analyse in which proteasome complexes the analysed structural subunits are integrated, 2D two-colour fluorescent immunoblot analysis was performed on liver lysates of naïve and infected WT (F) and *lmp7^−/−^* mice (G). Brackets mark the areas, in which the different proteasome fractions are found. qPCR analysis on total liver cDNA was performed to assess the mRNA expression of α3 (H) and α4I during the course of listeria infection in WT and *lmp7^−/−^* mice. The relative mRNA expression of the proteasome subunits was calculated by normalization to the housekeeping gene RPS9 using the ΔΔCT method. Each value represents mean ± standard deviation of three individual mice.

Expression of LMP7 accelerates proteasome maturation [17], but it is not clear how this function affects the proteasome system in infection. To analyse this, we quantified the abundance of various structural proteasome subunits in listeria-infected WT and *lmp7^−/−^* mice during the course of infection. As a correlate of proteasome quantity, antibodies recognizing multiple subunits (pan 20S-subunits and pan α-subunits; Fig. 6B, C) or antibodies specific for α3 and α4 were used (Fig. 6D, E). In livers of infected WT mice, we found increased abundance of all analysed proteasome subunits, which was highest at day 4 after listeria-infection (Fig. 6B–E). In contrast, proteasome subunits were not up-regulated in infected *lmp7^−/−^* mice. This resulted in a significantly higher abundance of all analysed subunits in WT mice throughout the course of infection (Fig. 6B–E). 2D Blue Native/SDS-PAGE confirmed that all proteasome subunits detected in WT mice (Fig. 6A), were integrated in mature proteasome complexes (Fig. 6F), demonstrating that the abundance of these subunits determined by immunoblot analysis serves as a suitable correlate for the amount of proteasomes. In conclusion, infection induced an increase in the quantity of mature proteasome complexes in WT but not *lmp7^−/−^* liver.

Interestingly, mRNA expression of α3 and α4 in the liver of WT mice was not induced by listeria-infection (Fig. 6 H, I), suggesting that the increased abundance of these subunits on protein level was caused by accelerated integration and stabilization in mature proteasome complexes of infected WT mice.

Similar results were obtained for the spleen, in which the abundance of all proteasome subunits was increased upon infection of WT, but not *lmp7^−/−^* mice (Fig. S5A–I). The major difference to the liver was that the abundance of all subunits was already higher in naïve WT mice as compared to *lmp7^−/−^* mice (Fig. S5B–E). This might be explained by the high basal expression of LMP7 in WT spleen, which is likely to promote accelerated proteasome maturation also in steady state lymphoid tissue.

To verify that the observed up-regulation of proteasome quantity is not unique to listeria-infection, the abundance of the structural proteasome subunits was further quantified in livers and spleens of WT and *lmp7^−/−^* mice infected with Lymphocytic choriomeningitis virus (LCMV), which is known to induce immunosubunit expression [22]. Comparable to listeria-infection, increased abundance of all analysed subunits was detected in both organs of WT, but not *lmp7^−/−^* mice (Fig. S6A–B). This demonstrates that the LMP7-dependent up-regulation of proteasome quantity also occurs after viral infection, suggesting that it is a general mechanism, which enhances the activity of the proteasome system in infection.

## Discussion

It has been shown that proLMP7 can mediate effective integration into various proteasome complexes, but is especially required for the maturation of LMP2/MECL-1-containing precursor proteasomes, which is known as model of cooperative immunoproteasome assembly (17, 18). In contrast, proβ5 has been suggested to mediate preferential formation of constitutive proteasomes due to specific interactions with β1/β2-containing precursor proteasomes. Accordingly, formation of mixed LMP2/MECL-1/β5 proteasomes is regarded as a rare event even in the absence of LMP7 [19]. Here, we analysed the impact of LMP7 and β5 on proteasome composition and maturation under inflammatory conditions and found that proLMP7- and proβ5-containing subunits integrated predominantly into LMP2/MECL-1-containing precursors. Thus proβ5 does not seem to mediate preferential integration into constitutive proteasomes under inflammatory conditions. Since this was confirmed in listeria-infected *lmp7^−/−^* mice, we conclude that in infection and inflammation, in which expression of LMP2 and MECL-1 is strongly induced, none of the propeptides mediates cooperative assembly of certain proteasome subtypes.

Our findings differ from reports defining the rules of cooperative assembly, which might be explained by the different approaches. While Griffin and Kingsbury et al. analysed cell lines in the steady state with fixed expression of constitutive and immunosubunits [18], [19], we used inducible systems, in which expression of LMP2 and MECL**_1_** is strongly up-regulated by IFNγ stimulation or infection. The strong induction of immunosubunits might have shifted the balance considerably towards formation of mixed LMP2/ MECL**_1_**/β5 proteasomes as compared to studies using fixed subunit expression. This is in line with work by Früh et al, who similar to our study employed inducible systems for the expression of immunosubunits and demonstrated that the expression level substantially influences the integration of immunosubunits. Hence, they suggested that integration of constitutive or immunosubunits is regulated by competition at the protein level [24]. In addition, they describe that integration of LMP2 and LMP7 can occur independent of each other, consequently resulting in any combination of β1 or LMP2 with β5 or LMP7, which is also in conflict with the rules of cooperative assembly [24]. In accordance, we found that β5 has a high capacity to integrate into mixed proteasomes with LMP2 and MECL-1, if competition with LMP7 is abrogated in mutant cell lines or mice, supporting that integration of constitutive or immunosubunits is rather regulated by competition at protein level than by cooperative assembly.

A further explanation for the conflicting data might be the different origins of the cell lines used to study the impact of immunosubunit on proteasome composition. We and Früh et al. used fibroblasts [24], which display high expression of constitutive subunits in the steady state, but strongly induced expression of immunosubunits upon IFNγ stimulation. In contrast, the data suggesting cooperative assembly were derived from T2 cells, which are of lymphoid origin and presumably express low levels of constitutive subunits [18], [19]. However, the expression level of β5 has not been defined in T2 cells and thus low formation of mixed LMP2/MECL-1/β5 proteasomes might be a result of restricted availability of β5 in this lymphoid cell line. In line with this, we found that formation of mixed LMP2/MECL-1/β5 proteasomes is less efficient in the spleen of *lmp7^−/−^* mice, which mostly contains lymphoid cell types, as compared to the liver, in which the tissue largely consists of non-lymphoid cells.

It has been proposed that proLMP7 is required for accelerated proteasome maturation mediated byLMP7, due to its high affinity to the proteasome maturation factor POMP [17], but direct experimental evidence was missing. Moreover it has been described that the catalytic activity of β-subunits is crucial to complete proteasome assembly [6], [9] and that carboxy-terminal domains of some β-subunits can have stabilizing effects, which facilitate proteasome maturation [25], [26]. Accordingly, each of these factors could contribute to accelerated proteasome maturation mediated by LMP7. Here we found that the fusion protein proLMP7mβ5 is equally efficient in mediating proteasome maturation as compared to full-length LMP7. Hence the proteolytic activity of β5 is not limiting for proteasome maturation and we can rule out that the specific catalytic activity of LMP7 is required to accelerate proteasome assembly. Moreover the proLMP7mβ5 fusion protein reveals that the carboxy-terminus of LMP7 is not required for accelerated proteasome maturation. These findings clearly identify proLMP7 as critical pacemaker, which drives accelerated proteasome maturation following induction of LMP7.

In yeast it has been shown that the propeptide of the β5 homologue Doa3 can support proteasome assembly, if it is expressed *in trans*, suggesting that it serves as an intra-molecular chaperone in the full-length protein [9]. In mammalian cells, we found that subunits containing proLMP7 have a 3 to 4-fold higher capacity to promote proteasome maturation in comparison to subunits containing proβ5, revealing that proLMP7 has a substantially higher chaperone activity as compared to proβ5. With regard to the regulation of proteasome composition, the high chaperone activity of proLMP7 constitutes a major advantage and is consistent with the fact that β5 is rapidly replaced, as soon as LMP7 is expressed [22], [23], [24]. This also explains, why mixed proteasomes with LMP2/β2/LMP7 or β1/β2/LMP7 stoichiometry can even be found during low-level expression of LMP7 in the steady state. Such mixed proteasomes were recently identified in various human tissues and cancer-derived cell lines and have been shown to be crucial for the MHC class I restricted presentation of specific tumour-derived peptides [20]. This clearly shows that the formation of these mixed proteasomes is not simply a bystander product of “unbalanced” expression of constitutive or immunosubunits, but that the chaperone function of proLMP7 is a relevant biological function.

In contrast, the low chaperone activity of proβ5 is a limiting factor for proteasome assembly under inflammatory conditions, resulting in accumulation of precursor proteasomes, if LMP7 is deleted. Moreover, the low chaperone activity of proβ5 is in line with the low abundance of LMP2/MECL-1/β5 proteasomes, when LMP7 is present. Still, we were able to detect LMP2/MECL-1/β5 proteasomes in WT-Mefs, however only, if the capacity of β5 to compete with LMP7 was increased by exogenous over-expression. Although this situation might be artificial, this finding is still mechanistically relevant, since it further underlines that integration of LMP7 and β5 is substantially regulated by their expression level and competition at the protein level. However, in this competition LMP7 has a substantial advantage due to its propeptide, which displays a higher affinity to POMP [17] and a superior chaperone activity as compared to proβ5. This does not only explain the rapid formation of immunoproteasomes in infection and inflammation, but is also consistent with the fact that LMP2/MECL-1/β5 proteasomes are rarely found in a normal wild-type situation.

Noteworthy, the proteasome content in *lmp7^−/−^* mice remained constant throughout the course of infection. This is in line with the low chaperone activity of proβ5 shown here and the approximately 4-fold lower rate of proteasome assembly mediated by β5 described by others [17]. It suggests that the physiological role of β5 is to provide a constant low-level turnover of proteasomes in the steady state. In this respect, the low chaperone activity of proβ5 is reasonable, as it appears to be adjusted to the long half-life, which has been reported for constitutive proteasomes [17].

Infection with *L. monocytogenes* or *Lymphocytic choriomeningitis virus* induces rapid replacement of constitutive by immunoproteasomes [22], [23] and accelerated proteasome assembly mediated by LMP7 supports this process [17]. However, beyond this mere shift to immunoproteasome formation, we found that induction of LMP7 leads to increased total proteasome quantity within the infected tissue. Hence, we identified a novel role of LMP7 in regulating the proteasome system by enhancing the output of active proteasome complexes. Still, the possibility remains that increased proteasome quantity of infected tissues in WT mice is a consequence of higher influx of immune cells as compared to *lmp7^−/−^* mice. This however is unlikely, since *lmp7^−/−^* mice develop normal, have unaltered numbers of T and B cells [12] and generate similar frequencies of listeria-specific CD8^+^ T cells as compared to WT mice [23].

Expression of LMP7 has been shown to promote inflammatory responses, which is in part due to improved NF-κB activation by LMP7-containing proteasomes [13], [14], [15]. Further, expression of LMP7 has been shown to facilitate degradation of oxidatively damaged proteins [16] and MHC class I antigen presentation [12]. So far, these functions of LMP7 have been exclusively associated with its proteolytic activity. Here, we show that optimal MHC class I antigen presentation was equally dependent on efficient maturation of proteasomes mediated by proLMP7. Thus it is tempting to speculate that the increase in proteasome quantity mediated by proLMP7, does not only improve MHC class I presentation, but might also be relevant for NF-κB activation and resistance to oxidative stress. Indeed, an increase in proteasome quantity mediated by the NFR2/KEAP-1 signalling pathway or the transcription factor NCF11 is known to enhance the resistance towards oxidative stress [27], [28]. At the same time, oxidative stress has been shown to induce LMP7 expression [29]. Thus it is likely that the LMP7-mediated increase in proteasome quantity protects against oxidative stress, either by itself or synergistically with other factors like e.g. NRF2/KEAP-1 and NCF11.

In summary, we describe a novel pathway of LMP7-mediated proteasome regulation, which relies on increased output of active proteasome complexes during infection and probably other inflammatory conditions. Moreover, we could identify proLMP7 as the critical pacemaker driving accelerated maturation of proteasomes in this context. Although it is likely that this mechanism contributes to the defence against infections, its precise impact on distinct biological functions such as MHC class I presentation, NF-κB activation and oxidative stress responses, remains a matter of future studies.

## Materials and Methods

### Experimental animals

All mice were kept under specific pathogen-free conditions (SPF). C57Bl/6N (WT) mice were obtained from Charles River (Berlin, Germany) and *lmp7^−/−^* mice were bred at the Max Planck Institute for Infection Biology (Berlin, Germany). Mice were infected intravenously (i.v.) with 5×10^3^ colony forming units (cfu) of *Listeria monocytogenes* strain EGD or 10^6^ pfu of *Lymphocytic choriomeningitis virus* strain WE (LCMV WE). All experiments were performed in strict accordance with German Animal Protection Law and granted by the ethical committee of the “Landesamt für Gesundheit und Soziales Berlin” (permission numbers G0165/04 and T0144/05) in Berlin, Germany.

### Cell cultures

Phoenix E cells, for ecotropic packaging of retroviral vectors (ATCC product # SD 3444), were kept in D10 Medium [Dulbeccós Modified Eagle Medium (Gibco) with 10% fetal calf serum (FCS), 1 mM L-glutamine, 1 mM sodium-pyruvate, 1× pencillin/streptomycin solution (Gibco), 50 µM β-mercaptoethanol]. Primary murine embryonic fibroblasts (Mefs) were isolated from 13- to 14-day-old embryos of WT or *lmp7^−/−^* mice. Spontaneous immortalization of Mefs was achieved by frequent passaging.

### Preparation of protein lysates

Frozen organs or cells were homogenized with a pestle. The homogenates were resuspended in 1 volume of 1×NativePAGE™ Sample Buffer (Invitrogen) complemented with 0.5% Igepal, 0.2 mM sodium vanadate, 5 mM sodium fluoride, 1 mM PMSF, 1 mM Pefabloc® SC (Roche Applied Science), 1× complete protease inhibitor cocktail (Roche Applied Science) and subjected to three freeze-thaw cycles. The debris was sedimented at 13,000 rpm for 30 min at 4°C. Protein concentrations were determined with Protein-Assay solution (Bio-Rad) according to manufacturer's instructions.

### Two-colour fluorescent immunoblot analysis

Twenty-five to fifty µg total protein diluted in 1× Laemmli buffer (50 mM TrisHCl pH6.8, 100 mM dithiothreitol, 2% (w/v) sodium dodecyl sulfate (SDS), 10% (v/v) glycerol, 0,1% (w/v) Bromophenol Blue) per lane were loaded on tris-glycine buffered 15% (w/v) SDS-PAGE gels and run in tris-glycine buffer (25 mM Tris, 250 mM Glycine, 0.1% (w/v) SDS). Following SDS-PAGE, proteins were transferred to Immobilon-FL PVDF membrane. Membranes were blocked in Odyssey Blocking Reagent (Licor Bioscience) and stained with rabbit or chicken polyclonal antibodies against proteasome subunits or POMP as well as mouse monoclonal antibodies against GAPDH. This was followed by staining with the respective secondary antibodies goat anti-rabbit IgG AlexaFluor680 (Molecular Probes), goat anti-chicken IgG IrDye700 (Rockland) or goat-anti-mouse IgG IrDye800 (Rockland). For evaluation, membranes were scanned with the Odyssey® Infrared Imaging system (Licor Biosciences). Densitometric analysis was performed with the Odyssey® Image Analyser Software Version 1.2 (Licor Biosciences). Band intensities were first normalized to the signal of the loading control GAPDH and subsequently to the signal of naïve WT mice (WT day 0), which was used as an internal standard on each gel.

Polyclonal rabbit antisera against β5, LMP2 as well as polyclonal chicken antibodies against β2 and POMP were obtained from Abcam. Rabbit polyclonal antibodies specific for MECL-1 were obtained from Biomol. The mouse monoclonal antibodies against α-subunits (MCP231), α3 (clone MCP257) and GAPDH (clone 6C5) were obtained from Calbiochem. Rabbit polyclonal antibodies against β1, LMP7, 20S proteasome subunits (MP3), α4, PA28αand 19S subunit S4 were kindly provided by the Institute for Biochemistry, Charité, Berlin.

### 2D Two-colour fluorescent immunoblot analysis

Organ lysates (50 µg per lane) supplemented with 0.125% (v/v) NativePAGE™ G-250 Sample Additive (Invitrogen) were loaded on NativePAGE™ Novex 4–16% Bis-Tris Gels (Invitrogen) and gels were run according to manufactureŕs instructions. Gels were sliced into single lanes and equilibrated in 2× Laemmli buffer for 30 min. Slices were placed in preparative slots of tris-glycine-buffered 15% (w/v) SDS-PAGE gels and run in tris-glycine buffer, which was followed by two-colour fluorescent immunoblot analysis (see above).

### Semiquantitative real-time RT-PCR (qPCR)

Organs were homogenized in TRIzol® Reagent (Invitrogen) and purification of RNA was performed according to manufacturer's instructions. The concentration and quality of the RNA was determined using a 2100 Bioanalyzer (Agilent Technologies). For cDNA synthesis, 2–4 µg total RNA were transcribed using random hexamer primers and SuperScript™ II Reverse Transcriptase (Invitrogen) according to manufacturer's instructions. qPCR reactions contained 1× SYBR Green mix (Applied Biosystems), 10 pmol forward-primer, 10 pmol reverse-primer and 5 µl cDNA template diluted 1∶20 in UltraPure Water (Millipore). The amplification was performed with an ABI Prism 7900H detection system (Applied Biosystems) and data were evaluated with the SDS2.2.2 Software (Applied Biosystems). The expression of ribosomal protein subunit 9 (RPS9) was used as internal standard. The relative expression was calculated as fold difference to RSP9 using the ΔΔCT method. Primer sequences are listed in Table S1.

### Generation of retrovirally transduced MEFs

The coding sequences of proβ5, proLMP7, β5, mβ5, LMP7 and mLMP7 with a carboxy-terminal Flag-tag fusion were amplified from a murine liver cDNA by nested PCR. The β5- and LMP7-Flag inserts were amplified using the primer pairs β5-start-for/β5-Flag-rev or LMP7-start-for/LMP7-Flag-rev. The proβ5mLMP7 or proLMP7mβ5 were generated by blunt-end ligation of the respective fragments using T4 Ligase (Fermentas) at 16°C overnight. The fragments were amplified using the following primer pairs: β5-start-for/proβ5-rev for proβ5, LMP7-start-for/proLMP7-rev for proLMP7, mβ5-for/β5-Flag-rev for mβ5 and LMP7-start-for/mLMP7-rev for mLMP7. All inserts were then amplified with the attB1- and attB2-adapter primers introducing the full attachment sites for Gateway recombination. The IRES-eGFP coding sequence was amplified with the primers IRES-eGFP-for/IRES-eGFP-rev containing the attB2/attB3 attachment sites and the EF1α-Promotor was amplified with primers EF1α-for-B4/EF1α-rev-B1 containing the attB4/attB1 attachment sites. Primer sequences are listed in Table S1. The proteasome subunit inserts were first subcloned into the entry vector pDONR™221 (Invitrogen), the IRES-eGFP insert into pDONR™P2R-P3 (Invitrogen) and the EF1α-promotor into pDONR™P4-P1R using the BP-recombination reaction according to the MultiSite Gateway® Three-Fragment Vector Construction Kit Manual. Final expression vectors were generated by a LR-recombination reaction between pDest-Super, pDONR™P4-P1R-EF-1α, the pDONR™221 entry vectors containing the proteasome-subunit inserts and pDONR™P2R-P3-IRES-eGFP according to manufacturer's instructions resulting in the expression vectors pEX-EF-1α-β5-Flag-IRES-eGFP, pEX-EF-1α-LMP7-Flag-IRES-eGFP, pEX-EF-1α-proLMP7mβ5-Flag-IRES-eGFP and pEX-EF-1α-proβ5mLMP7-Flag-IRES-eGFP, respectively. The expression vectors were packed into ecotropic retroviral particles by transient transfection of Phoenix E cells using CaPO_4_ precipitation. Twenty-four hours after transfection the medium was exchanged and the supernatants containing retroviral particles were harvested after 24 and 48 h. For retroviral transduction 2 ml of the supernatants were added per well to Mefs seeded in 6-well plates with 50% confluency. The supernatants were replaced by fresh supernatant 24 h later and then exchanged against D10 medium supplemented with 10 µg/ml puromycin (Sigma-Aldrich) another 24 h later for selection. After 2–4 weeks, eGFP-expressing cells were repeatedly sorted with a DIVA cell sorter (BD Biosciences) until a purity of >98% of eGFP high-expressing Mefs was achieved.

### Co-immunoprecipitation

Retrovirally transduced *lmp7^−/−^* Mefs expressing β5-Flag, LMP7-Flag, proLMP7mβ5-Flag and proβ5mLMP7-Flag or containing an empty eGFP-expression vector were either left untreated or stimulated with 100 U/ml IFNγ(Strathmann Biotec). After 4 days the cells were harvested and cell-lysates were prepared according to the instruction described above. Cell-lysates (600 µg total protein) were diluted in 300 µl lysis buffer (50 mM TrisHCl pH 7.4, 150 mM NaCl, 1mM EDTA, 0,5% Igepal) and mixed with 80 µl Anti-Flag® M2 Affinity Gel (Sigma Aldrich) equilibrated in TBS (25 mM TrisHCl pH 7.4, 50 mM NaCl). Samples were shaken head over tail at 4°C over night and then the gel matrix was sedimented at 8,000 × *g* for 1 min. The supernatant was saved and mixed with 1 volume 2× Laemmli buffer. The gel matrix was washed thrice with 0.5 ml TBS and subsequently resuspended in 300 µl lysis buffer plus 300 µl 2× Laemmli buffer. The supernatants and the precipitated proteins (pull down) were denaturized at 95°C for 5 min and 25 µl/lane, resembling 25 µg of the initial total protein input, were loaded on 15% SDS-PAGE gels.

### Measurement of MHC class I cell surface expression

*lmp7^−/−^* Mefs expressing β5-Flag, LMP7-Flag, proLMP7mβ5-Flag and proβ5mLMP7-Flag or the empty eGFP-expression vector were grown in the presence of 50 U/ml IFNγ for 4 days. Cells (1×10^6^) were stained in FACS-buffer (PBS, 0.1% BSA, 2 mM NaN_3_) containing the haplotype-specific antibodies H-2K^b^-APC (clone AF88.5.5.3, BD Biosciences) or H-2D^b^-Cy5 (clone HB27) at 4°C for 30 min. Cells were analysed on a FACS-Canto (BD Biosciences). The relative MHC class I surface density of H-2K^b^ and H-2D^b^ was determined as median fluorescence intensity (MFI).

### Statistics

All values presented in figures throughout the manuscript are means ± standard deviation. The number of replicates for each experiment is given in the figure legend. Significance was determined by unpaired, two-tailed t test and the indicated significance levels are n.s. – not significant, * P<0.05, ** P<0.01, *** P<0.001.

## Supporting Information

Figure S1 **Co-immunoprecipitation analysis in** ***lmp7 ^−/−^*** **Mefs transduced with the empty vector construct and in WT-MEFs overexpressing β5.**
(PDF)

Figure S2 **Identification of mature proteasome and precursor complexes by 2D two-colour fluorescent immunoblot analysis.**
(PDF)

Figure S3 **Analysis of proteasome composition in naive and listeria-infected WT mice.**
(PDF)

Figure S4 **Quantifying the expression of β1, β2, LMP2 and MECL-1 at protein and mRNA level in WT and** ***lmp7^−/−^*** **mice during the course of listeria infection.**
(PDF)

Figure S5 **Relative Quantification of mature proteasomes in WT and** ***lmp7^−/−^*** **mice during the course of listeria infection.**
(PDF)

Figure S6 **Relative quantification of proteasome abundance in WT and** ***lmp7^−/−^*** **mice during the course of LCMV infection.**
(PDF)

Table S1 **Primer sequences used for qPCR and molecular cloning.**
(PDF)
