# Supplementary material for: The Proteasome System in Infection: Impact of β5 and LMP7 on Composition, Maturation and Quantity of Active Proteasome Complexes
Source: PLoS One. 2012 Jun 29;7(6):e39827. doi: 10.1371/journal.pone.0039827 (PMC3387237; doi:10.1371/journal.pone.0039827)
Supplement: Figure S1 — Co-immunoprecipitation analysis in lmp7 −/− Mefs transduced with the empty vector construct and in WT-MEFs overexpressing β5. (PDF) [file pone.0039827.s001.pdf]

FIGURE S1

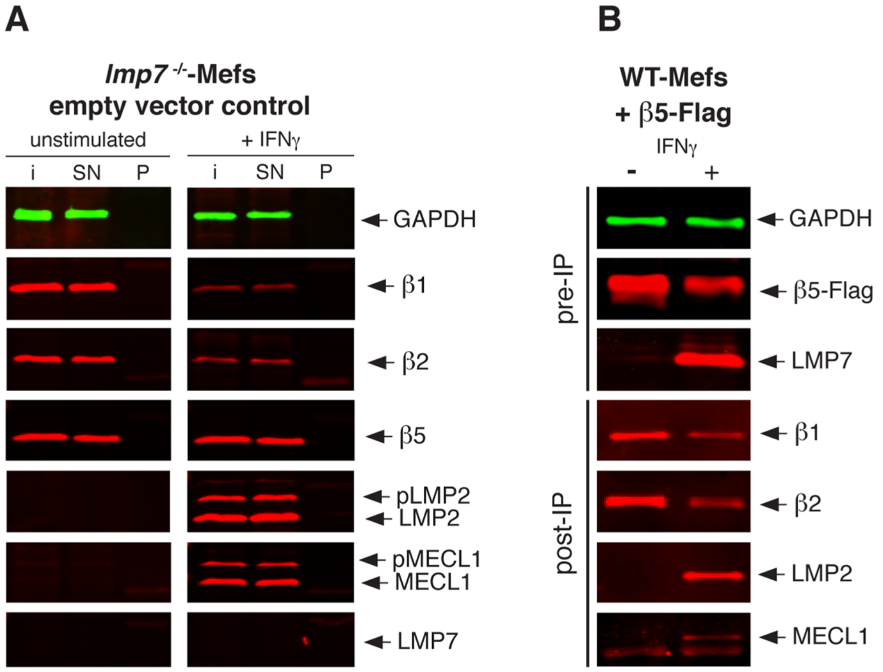

Figure S1: Co-immunoprecipitation analysis in *lmp7* $^{-/-}$ Mefs transduced with the empty vector construct and in WT-MEFs overexpressing β5

(A) *lmp7*$^{-/-}$ Mefs were transduced with the empty vector, which was used for retroviral transduction of the *lmp7*$^{-/-}$ Mefs expressing LMP7-Flag and proLMP7mβ5-Flag or the proβ5-containing subunits β5-Flag and proβ5mLMP7-Flag (Figure 1A). The *lmp7*$^{-/-}$ Mef with the empty vector were either left unstimulated or cultured in the presence of 50 U/ml IFNγ for 4 days, which was followed by co-immunoprecipitation analysis to control that the precipitation shown in Figure 1 was specific for Flag-tagged proteasome complexes. Two-colour fluorescent immunoblot analysis confirmed that the precipitates (P) were negative for β1, β2, β5, LMP2, MECL-1 and LMP7, and that all non-Flag-tagged proteasome from the input material (i) were recovered in the supernatants (SN). (B) WT-Mefs were retrovirally transduced to overexpress Flag-tagged, full-length β5 (β5-Flag). WT-Mefs + β5-Flag were either left untreated or cultured in the presence of 50 U/ml IFNγ for 4 days, which was followed by co-immunoprecipitation analysis. The abundance of GAPDH, β5-Flag and LMP7 was checked in the input material (pre-IP) and the abundance of β1, β2, LMP2 and MECL-1 in the precipitate (post-IP).
