# Supplementary material for: The Proteasome System in Infection: Impact of β5 and LMP7 on Composition, Maturation and Quantity of Active Proteasome Complexes
Source: PLoS One. 2012 Jun 29;7(6):e39827. doi: 10.1371/journal.pone.0039827 (PMC3387237; doi:10.1371/journal.pone.0039827)
Supplement: Figure S2 — Identification of mature proteasome and precursor complexes by 2D two-colour fluorescent immunoblot analysis. (PDF) [file pone.0039827.s002.pdf]

FIGURE S2

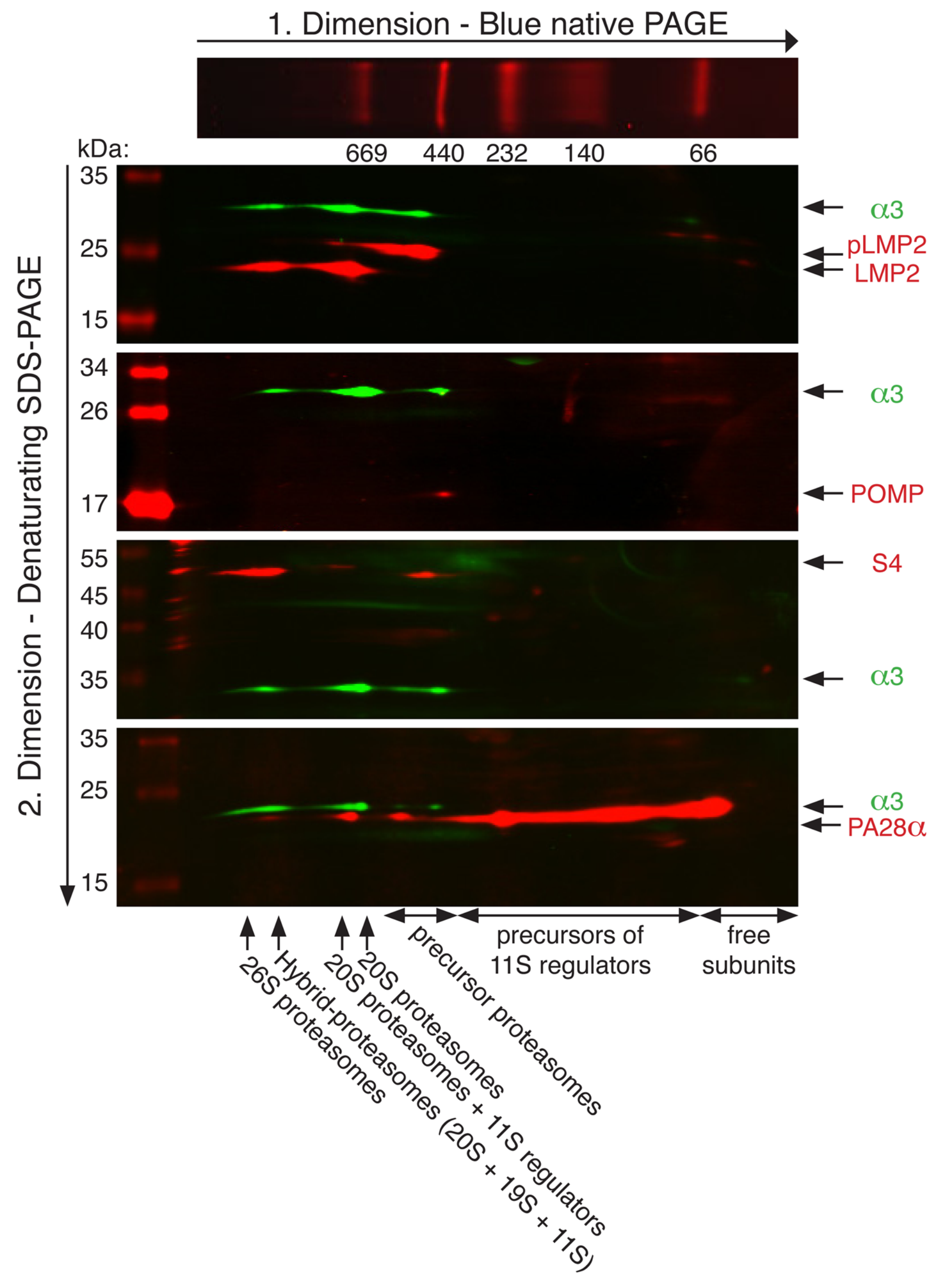

Figure S2: Identification of mature proteasome and precursor complexes by 2D two-colour fluorescent immunoblot analysis

Organ-lysates of spleens of *lmp7*$^{-/-}$ mice, which were infected i.v. with 5 × 10^{3} cfu of L. monocytogenes 4 days before were analysed by 2D two-colour fluorescent immunoblot analysis. First, protein complexes were separated by Blue Native-PAGE and in the second dimension subjected to SDS-PAGE, which was followed by two-colour fluorescent immunoblot analysis. Each membrane was stained for α3 (green signal) and as it is present in all early to mature complexes in proteasome assembly, α3 serves as a marker for the presence and positions of 13-15S precursor proteasomes, 20S proteasomes and 20S proteasomes + 11S and 19S regulators complexes. Staining for known components identified the various complexes: 13-15S precursor proteasomes were identified by the presence of unprocessed LMP2 (pLMP2) and POMP, 19S regulators by presence of ATPase subunit S4 and 11S regulators by staining for PA28α (red signals). Arrows indicate the positions of the various identified proteasome complexes. Organs of three to four mice per group were pooled for the analysis.
