# Supplementary material for: The Proteasome System in Infection: Impact of β5 and LMP7 on Composition, Maturation and Quantity of Active Proteasome Complexes
Source: PLoS One. 2012 Jun 29;7(6):e39827. doi: 10.1371/journal.pone.0039827 (PMC3387237; doi:10.1371/journal.pone.0039827)
Supplement: Figure S3 — Analysis of proteasome composition in naive and listeria-infected WT mice. (PDF) [file pone.0039827.s003.pdf]

FIGURE S3

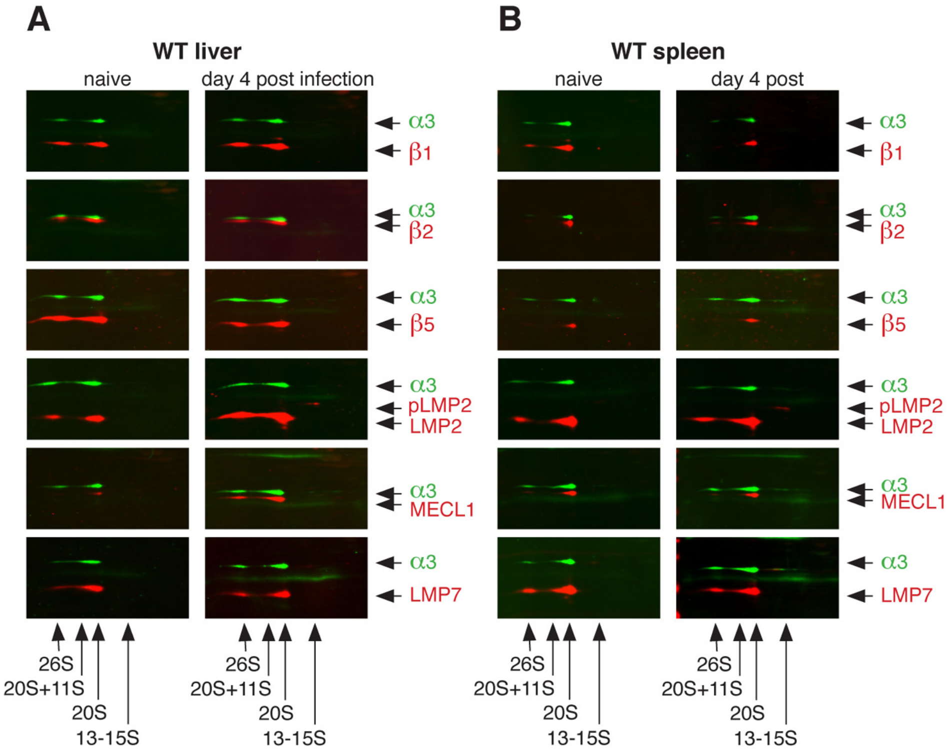

Figure S3: Analysis of proteasome composition in naive and listeria-infected WT mice

Organ lysates of liver (A) and spleen (B) of naïve and infected WT mice were analysed by 2D two-colour fluorescent immunoblot analysis. The mice were infected i.v. with 5×10^3 cfu L. monocytogenes and sacrified 4 days post-infection. Organs of three to four mice per group were pooled for the analysis. The different proteasome complexes present in the tissues were first separated by Blue Native-PAGE and subsequently by SDS-PAGE followed by Two-colour fluorescent immunoblot analysis. Each membrane was stained for proteasome subunit α3 (green signal) and as it is present in all early to mature complexes in proteasome assembly, α3 serves as a marker for the presence and positions of 13-15S precursor proteasomes, 20S proteasomes and 20S proteasomes + 11S and 19S regulators complexes. Further, membranes were stained for the indicated catalytic β subunits (red signals) to identify, in which of the indicated proteasome complexes they were integrated.
