# Supplementary material for: The Proteasome System in Infection: Impact of β5 and LMP7 on Composition, Maturation and Quantity of Active Proteasome Complexes
Source: PLoS One. 2012 Jun 29;7(6):e39827. doi: 10.1371/journal.pone.0039827 (PMC3387237; doi:10.1371/journal.pone.0039827)
Supplement: Figure S4 — Quantifying the expression of β1, β2, LMP2 and MECL-1 at protein and mRNA level in WT and lmp7−/− mice during the course of listeria infection. (PDF) [file pone.0039827.s004.pdf]

FIGURE S4

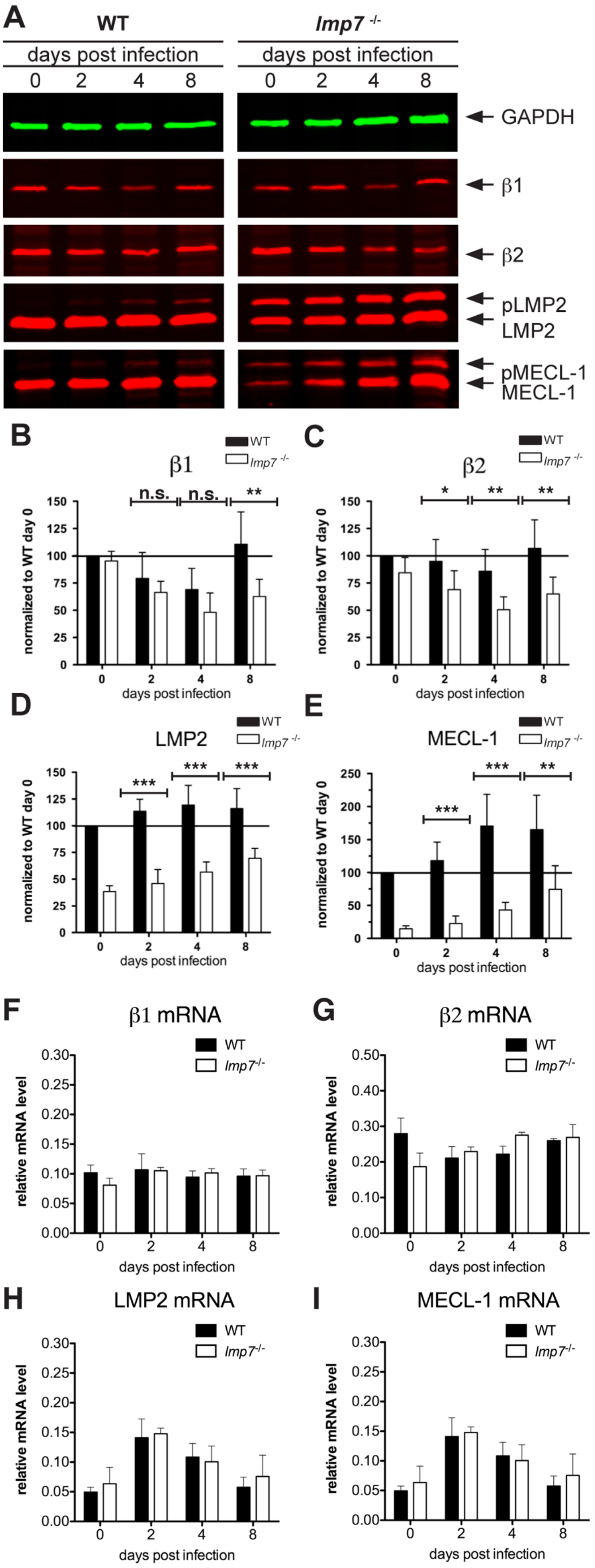

Figure S4: Quantifying the expression of β1, β2, LMP2 and MECL-1 at protein and mRNA level in WT and *lmp7* -/- mice during the course of listeria infection

Both groups of mice were infected i.v. with 5×10^3 cfu of listeria and spleens of three to four mice per group were pooled for two-colour fluorescent immunoblot analysis. Naïve mice (day 0) were used as controls in both groups of mice. Each membrane was stained against GAPDH as loading control. Further, membranes were stained with antibodies specific for β1, β2, LMP2 and MECL-1 as indicated. Representative blots of three independent experiments are shown (A). Following densitometric analysis the relative protein abundance of β1 (B), β2 (C), LMP2 (D) and MECL-1 (E) was expressed as band intensities normalized to WT day 0, which was calculated as follows: (band intensity of proteasome subunit X at day Y / band intensity of GAPDH at day Y)/(band intensity of protesome subunit X in WT day 0 / band intensity of GAPDH in WT day 0) × 100). The given results are means ± standard deviation of three independent infections and each sample was analysed in duplicates during immunoblot analysis (B-E). Total splenic RNA of naïve and listeria-infected WT and *lmp7*-/- mice was isolated at the indicated time points and semi-quantitative qPCR analysis was performed. The relative mRNA expression of the proteasome subunits β1 (F), β2 (G), LMP2 (H) and MECL-1 (I) were calculated by normalization to the house-keeping gene ribosomal protein subunit 9 (RPS9) using the ΔΔCT method. Each value represents mean ± standard deviation of three individual mice. Representative results of one of two independent infection experiments are shown.
