# Supplementary material for: The Proteasome System in Infection: Impact of β5 and LMP7 on Composition, Maturation and Quantity of Active Proteasome Complexes
Source: PLoS One. 2012 Jun 29;7(6):e39827. doi: 10.1371/journal.pone.0039827 (PMC3387237; doi:10.1371/journal.pone.0039827)
Supplement: Figure S5 — Relative Quantification of mature proteasomes in WT and lmp7−/− mice during the course of listeria infection. (PDF) [file pone.0039827.s005.pdf]

FIGURE S5

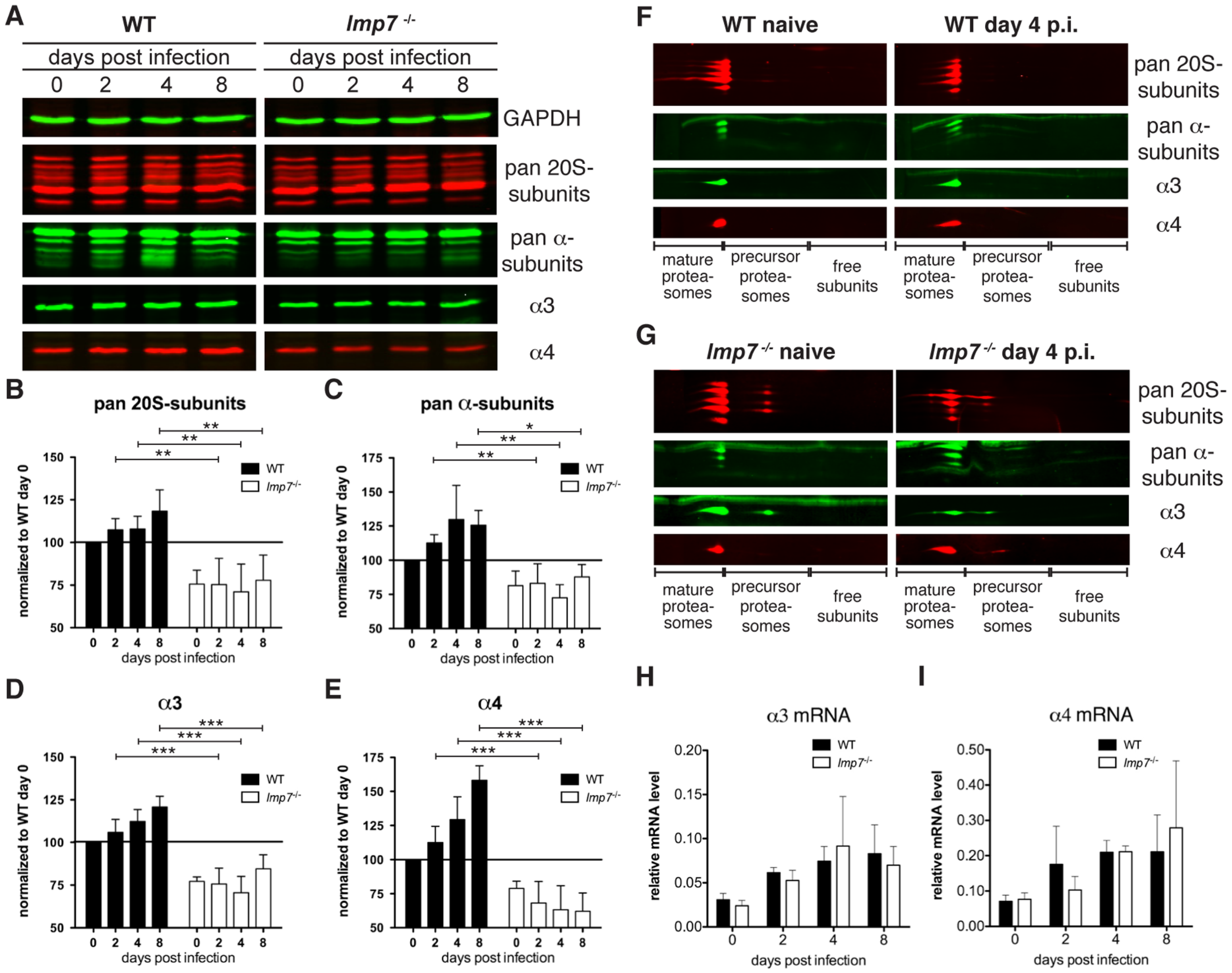

Figure S5: Relative Quantification of mature proteasomes in WT and ***lmp7*** $^{-/-}$ **mice during the course of listeria infection**

Both groups of mice were infected i.v. with 5×10^3 cfu of listeria and spleens of three to four mice per group were pooled for two-colour fluorescent immunoblot analysis. Naïve mice (day 0) were used as controls in both groups of mice. Each membrane was stained against GAPDH as loading control. Further, membranes were stained with antibodies recognizing pan-20S-subunits, pan-α-subunits, α3 and α4 as indicated. Representative blots of three independent experiments are shown (A). Following densitometric analysis the relative protein abundance of pan-20S subunits (B), pan-α subunits (C), α3 (D) and α4 (E) was expressed as band intensities normalized to WT day 0, which was calculated as follows: (band intensity of protesome subunit X at day Y / band intensity of GAPDH at day Y)/(band intensity of protesome subunit X in WT day 0 / band intensity of GAPDH in WT day 0) × 100). The given results are means ± standard deviation of three independent infection experiments and each sample was analysed in duplicates during immunoblot analysis (B-E). To analyse in which proteasome complexes the analysed structural subunits are integrated, 2D Two-colour fluorescent immunoblot analysis was performed on spleen lysates of naïve and infected WT (F) and *lmp7* $^{-/-}$ mice (G). Brackets mark the areas in which the different proteasome fractions are found. qPCR analysis on total splenic cDNA was performed to assess the mRNA expression of α3 (H) and α4 (I) during the course of listeria infection in WT and *lmp7* $^{-/-}$ mice. The relative mRNA expression of the proteasome subunits was calculated by normalization to the house keeping gene RPS9 using the ΔΔCT method. Each value represents mean ± standard deviation of three individual mice.
