# Supplementary material for: The Proteasome System in Infection: Impact of β5 and LMP7 on Composition, Maturation and Quantity of Active Proteasome Complexes
Source: PLoS One. 2012 Jun 29;7(6):e39827. doi: 10.1371/journal.pone.0039827 (PMC3387237; doi:10.1371/journal.pone.0039827)
Supplement: Figure S6 — Relative quantification of proteasome abundance in WT and lmp7−/− mice during the course of LCMV infection. (PDF) [file pone.0039827.s006.pdf]

FIGURE S6

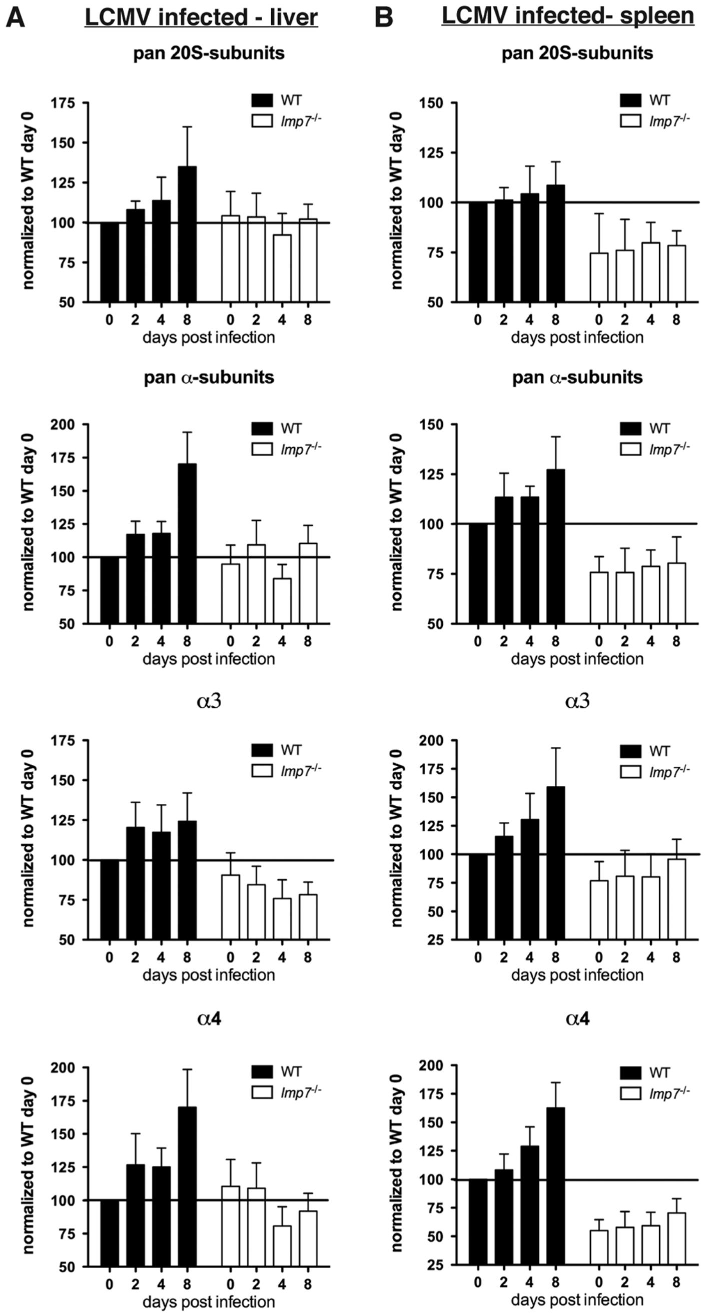

Figure S6: Relative quantification of proteasome abundance in WT and *lmp7* $^{-/-}$ mice during the course of LCMV infection

WT and *lmp7*$^{-/-}$ mice were infected i.v. with 10^6 pfu of LCMV strain WE and organs were taken at the indicated time points. Livers (A) and spleens (B) of three to four mice per group were pooled for Two-colour fluorescent immunoblot analysis. Naïve mice (day 0) were used as controls in both groups of mice and each membrane was stained against GAPDH as loading control. Following densitometric analysis the relative protein abundance of pan-20S-subunits, pan-α subunits, α3 and α4 was expressed as band intensities normalized to WT day 0, which was calculated as follows: (band intensity of protesome subunit X at day Y / band intensity of GAPDH at day Y)/(band intensity of protesome subunit X in WT day 0 / band intensity of GAPDH in WT day 0) × 100). The given results are means ± standard deviation of two infection experiments and each sample was analysed in duplicates during immunoblot analysis (A-B).
