# Supplementary material for: The Proteasome System in Infection: Impact of β5 and LMP7 on Composition, Maturation and Quantity of Active Proteasome Complexes
Source: PLoS One. 2012 Jun 29;7(6):e39827. doi: 10.1371/journal.pone.0039827 (PMC3387237; doi:10.1371/journal.pone.0039827)
Supplement: Table S1 — Primer sequences used for qPCR and molecular cloning. (PDF) [file pone.0039827.s007.pdf]

Table S1: Primer sequences used for qPCR and molecular cloning

primer	sequence (5' to 3')*
β1-for	TGACCAAGGACGAATGTCTG
β1-rev	GATTTGGTCTCCCAAAAGCA
β2-for	CTGTCTTGGAAGCGGATTTC
β2-rev	GCAACAACCATCCCTTCAGT
β5-for	GTGAATCAGCACGGGTTTT
β5-rev	AATCCGCTGCAACAATGACT
LMP2-for	CATCATGGCAGTGGAGTTTGAC
LMP2-rev	ACCTGAGAGGGCACAGAAGATG
MECL-1-for	CAGCCAAACATGACGCTGG
MECL-1-rev	CAGTGATCACACAGGCATCCAC
LMP7-for	ACCACACTCGCCTTCAAGTTC
LMP7-rev	GCCAAGCAGGTAAGGGTTAATC
POMP-for	AACATCCAGGGTCTGTTTGC
POMP-rev	TCGTTGCCCCTCAAAATATC
α3-for	TGCCATGAAGGCTGTGGAAAACA
α3-rev	ACGAGCATCTGCCAGCAAACC
α4-for	CGGAGAGGCGCAACATCCAC
α4-rev	TGTTATGCCTGCCACGCTGC
RPS9-for	CTGGACGAGGGCAAGATGAAGC
RPS9-rev	TGACGTTGGCGGATGAGCACA
β5-start-for	AAAAAGCAGGCTCCACCATGGCGCTGGCTAGCGTG
β5-Flag-rev	AGAAAGCTGGGTCTCAGATCTTATCGTCGTCATCCTTG-<br>TAATCGGGGACAGATACACTAC
mβ5-for	ACCACCACCCTGGCCTTC
proβ5-rev	TCCGTGAAGCATTTCGATTC
LMP7-start-for	AAAAAGCAGGCTCCACCATGGCGTTACTGGATCTGTG
LMP7-Flag-rev	AGAAAGCTGGGTCTCAGATCTTATCGTCGTCATCCTTG-<br>TAATCCAGAGCGGCCTCTCCG
mLMP7-for	ACAACCACACTCGCCTTCAAG
proLMP7-rev	GCCGTGGGCCATCTCAAT
attB1-for	GGGGACAAGTTTGTACAAAAAAGCAGGCT
attB2-rev	GGGGACCACTTTGTACAAGAAAGCTGGGT
IRES-eGFP-for	GGGGACAGCTTTCTTGTACAAAGTGGTCAGCTTCGAATTCTGCAGTCG
IRES-eGFP-rev	GGGGACAACTTTGTATAATAAAGTTGCTTACTTGTACAGCTCGTCCATGC
EF1a-for-B4	GGGGACAACTTTGTATAGAAAAGTTGCATGGCTCCGGTG<br>CCCGTCAGTGG
EF1a-rev-B1	GGGGACTGCTTTTTTGTACAAACTTGAACCTGAAATGG<br>AAGAAAAAAA

*All primers were obtained from TIB Molbiol, Berlin, Germany.
